# FOLFOXIRI plus panitumumab for left-sided RAS-wild-type metastatic colorectal cancer

**DOI:** 10.1093/oncolo/oyag320

**Published:** 2026-08-18

**Authors:** Howard S Hochster, Patrick J Blatchford, Stacey Stein, Jill Lacy, Anup Kasi, Patrick M Boland, Philp Gold, Aaron J Scott, Paul Oberstein, Michael Cusnir, Kate Rouse, Karen Mallalieu, Anwaar Saeed, For the AGICC Group

**Affiliations:** Rutgers Cancer Institute, New Brunswick, NJ 08901, United States; Blatchford Analytics, Denver, CO 80201, United States; Rutgers Cancer Institute, New Brunswick, NJ 08901, United States; Yale Cancer Center, New Haven, CT 06520, United States; University of Kansas, Kansas City, KS 66160, United States; Rutgers Cancer Institute, New Brunswick, NJ 08901, United States; Swedish Cancer Institute, Seattle, WA 98122, United States; University of Arizona, Tucson, AZ 85719, United States; NYU Perlmutter Cancer Center, New York, NY 10023, United States; Mt Sinai Medical Center, Miami, FL 33140, United States; Criterium Inc. Saratoga Springs, NY 12866, United States; Criterium Inc. Saratoga Springs, NY 12866, United States; UPMC, Pittsburgh, PA 15232, United States

**Keywords:** colorectal cancer, chemotherapy, RAS wild-type

## Abstract

**Background:**

Colorectal cancer (CRC) can be effectively treated with fluoropyrimidine chemotherapy doublets. The triplet chemotherapy program, FOLFOXIRI, is more effective than a doublet in randomized trials. We combined this triplet with the anti-epidermal growth factor receptor (EGFR) antibody, panitumumab, as first-line therapy in the appropriately targeted population: left-sided, RAS wild-type metastatic CRC.

**Methods:**

A multicenter trial was conducted with the AGICC (Academic GI Cancer Consortium) group. Patients with stage IV or unresectable, left-sided, Rat Sarcoma Virus oncogene (RAS) wild-type CRC were eligible if they had measurable disease, adequate organ function, and Perfromance Status (PS) 0-1. Consented patients were treated with modified FOLFOXIRI and panitumumab every 2 weeks until progression or toxicity. CT scans were performed every 8 weeks. The primary endpoint was response rate by Response Evaluation Criteria in Solid Tumors (RECiST) v1.1, with an expectation of an RR ≥85%.

**Results:**

Twenty-nine patients were enrolled and 27 were treated. A total of 430 cycles were given, including 7 patients with the maximum of 24 cycles (median 18, range 3-24). Best response included 2 CR, 18 PR, and 6 SD (RR 74%). One patient progressed at the time of first assessment. Grade 3+ toxicity included neutropenia, diarrhea, rash, hypomagnesemia, and hypokalemia, similar to prior reports. No patient had grade 5 toxicity. Median progression-free survival (PFS) was 17.0 months, and overall survival was not reached but exceeded 36 months.

**Conclusion:**

In this small US multicenter study, utilizing FOLFOXIRI with an anti-EGFR antibody for appropriately selected patients, we note that the response rates and PFS values are among the highest reported in stage IV colon cancer.

**ClinicalTrials.gov identifier:**

NCT04169347.

Lessons learned We report a multicenter US phase 2 study of first-line therapy with FOLFOXIRI and panitumumab for metastatic colorectal cancer in patients selected for maximal potential benefit (left-sided RAS/BRAF wild-type tumors).

- Confirmation of a high response rate (74%; 95% CI, 54-89%) for the regimen.
- Confirmation of prolonged PFS (17 months) and overall survival (>36 months).
- The regimen should be considered for appropriate patients with left-sided, RAS-wild-type colorectal cancer.

## Introduction

Colorectal cancer (CRC) remains the third leading cause of cancer death in the United States.^1^ Advances in combination chemotherapy over the past few decades have resulted in improved overall survival (OS) and progression-free survival (PFS) for patients with metastatic CRC.^2^ But despite 5-fluorouracil/folinic acid, oxaliplatin, irinotecan (FOLFOX, FOLFIRI), anti-vascular endothelial growth factor (VEGF), and anti-epidermal growth factor receptor (EGFR)-based therapies, the 5-year survival rate is <20%.^2–7^ Triplet therapy with FOLFOXIRI was found to have a higher Overall Response Rate (ORR), PFS, and OS than doublet therapy with FOLFIRI,^8^ with further improvement with the addition of bevacizumab.^9^

Additionally, primary tumor location affects outcomes in first-line therapy of metastatic CRC, with right-sided tumors having substantially shorter median survival compared with left-sided primary CRC. Retrospective analyses of large phase III trials comparing chemotherapy plus either anti-EGFR or anti-VEGF agents^10^^,^^11^ revealed that OS was longer for left-sided CRC (33.3 vs 19.4 months, *P* < .0001). In addition, cetuximab plus chemotherapy showed improved OS and PFS compared with bevacizumab for left-sided CRC (OS 36.0 vs 31.4 months; PFS 12.4 vs 11.2 months).^12^ In the Gruppo Oncologico del Nord Ovest (GONO) study,^13^^,^^14^37 patients were treated with FOLFOXIRI with an ORR of 89% (1 CR and 32 PR), a median PFS 11.3 months, and a median OS that was not reached with a 17.7 months follow-up. In the randomized phase 2 Arbeitsgemeinschaft Internistische Onkologie (AIO) trial of mFOLFOXIRI ± panitumumab,^15^ the RR for left-sided tumors was 91% vs 60% (as compared with 60% vs 50% for right-sided CRC).

Considering the results above, we conducted a multicenter US trial to evaluate the efficacy of FOLFOXIRI and panitumumab as first-line therapy for metastatic, left-sided, RAS wild-type CRC, the optimal subset for this regimen. No study had selected patients based on “sidedness” when we initiated this trial. Given the above, we wished to develop US data based on our practice patterns.

**Table:** 

**TRIAL INFORMATION**	
**Disease**	Metastatic colorectal cancer
**Stage of disease**	IV
**Prior therapy**	None (first-line therapy)
**Type of study**	Phase II
**Primary endpoints**	Objective RECIST response based on the patient’s best response
**Secondary endpoints**	Overall survival, progression-free survival, and safety
**NCT number**	04169347
**Additional details of endpoints or study design:** The study focused on the molecularly defined subset of Left-sided RAS wild-type colorectal cancer. The goal was to provide evidence of FOLFOXIRI and anti-EGFR activity in a multicenter US trial.	

## Methods

Patients were eligible with a histopathologic diagnosis of previously untreated metastatic CRC (in the case of prior adjuvant therapy, >6 months since completion required). Patients also had left-sided colorectal cancer and an extended RAS mutation analysis (including KRAS/NRAS exons 2, 3, and 4 and BRAF), without mutation by any CLIA-certified tissue or blood-based genomic profile. One interim cycle of FOLFOX or FOLFOXIRI prior to enrollment was allowed. Also required were an ECOG PS of 0 or 1, measurable disease by RECiST v1.1, and lab values had to be within usual trial parameters, including normal albumin. Exclusions included neuropathy of grade 2 or higher, known hypersensitivity to any of the treatments, or known DPD deficiency. The study was approved by the central IRB and local IRBs for participation and was opened in March 2020 (despite COVID-19). The study was closed to accrual on 30 June 2023 and formally closed on 31 December 2024. Participating institutions included the Rutgers Cancer Institute, University of Arizona, University of Kansas, Swedish Cancer Institute, Yale University, NYU Medical Center, and Mt Sinai (Miami) Medical Center. An independent Data Safety Monitoring Committee (DSMC) monitored study outcomes. The study was registered on ClinicalTrials.gov.

### Treatment

Panitumumab was given every 2 weeks over 60 min and then over 30 min subsequently, followed by chemotherapy. Chemotherapy included FOLFOXIRI: irinotecan 150 mg/m², followed by oxaliplatin 85 mg/m^2^, leucovorin 200 mg/m², and 5-FU 2400 mg/m² over 46-48 h. Pegfilgrastim 6 mg was given on day 3 as per the investigator’s discretion. Treatment was continued up to 24 cycles, until disease progression, excessive toxicity, physician decision, or patient request. Treatment was modified according to standard institutional guidelines for toxicity with specific panitumumab and oxaliplatin dose reductions, per respective package inserts. All patients signed informed consent per institutional guidelines and as approved by local Instituional Review Boards.

### Supportive care

All supportive care was allowed at the physician’s discretion. Doxycycline 100 mg qd and topical steroids were recommended.

### Statistics

CT scans were performed every 4 cycles for determination of response by RECiST 1.1 criteria. The primary endpoint was response rate using a Simon two-stage design with the hypothesis of at least an 80% response rate. Thirty-five patients were to be enrolled with early stopping for fewer than 18 responses in the first 27 patients evaluated. The null hypothesis would be rejected if fewer than 25 of 35 patients responded, with a type 1 error of 0.1 and power of 90% to detect a true response rate of 80% or higher. The probability of stopping early was 82%. An expected sample size of 29 patients was sufficient for determination of the primary hypothesis of RR >80%. PFS and OS were determined by the Kaplan-Meier method. We were required to end the study early due to funding considerations with 29 patients accrued, including 27 evaluable for response.

## Results

Forty-one patients were consented for screening, of whom 29 were eligible and enrolled. However, two of these patients did not receive any treatment, so they were not considered in the analysis cohort for reporting results. The subject characteristics are shown in the table above, including a mean age of 51.4 years (range 38-76).

**Table:** 

**PATIENT CHARACTERISTICS, *N* = 27 patients**	
**Gender**	
**Male**	20 (74%)
**Female**	7 (26%)
**Stage IV**	27 (100%)
**Number of prior systemic therapies: median (range)**	0
**Performance status: ECOG 0/1**	19/8 (70/30%)
**Ethnicity**	0
**Hispanic or Latino**	4 (15%)
**Non-Hispanic or Latino**	22 (81%)
**Unknown**	1 (4%)
**Race**	
**Caucasian**	24 (89%)
**African American**	0
**Asian**	2 (7%)
**Native Hawaiian or Pacific Islander**	1 (4%)
**Age**	
**Mean (range), years**	51 (38-76)
**Median (Q1-Q3), years**	50 (46-58)

**Table:** 

**PRIMARY ASSESSMENT METHOD**	
**Evaluation method**	RECIST 1.1
**Number of patients enrolled**	29
**Number of patients evaluable**	27
**Endpoints**	ORR, DCR, PFS, OS
**Response assessments**	
**CR**	2 (7%)
**PR**	18 (67%)
**SD**	6 (22%)
**PD**	1 (4%)
**ORR**	20 (74%)
**DCR**	26 (96%)
**Response duration**	
**PFS (median)**	17.0 months (95% CI, 13- Not Reached (NR))
**OS (median)**	>35.4 months (NR)

### Response

**Figure 1.**
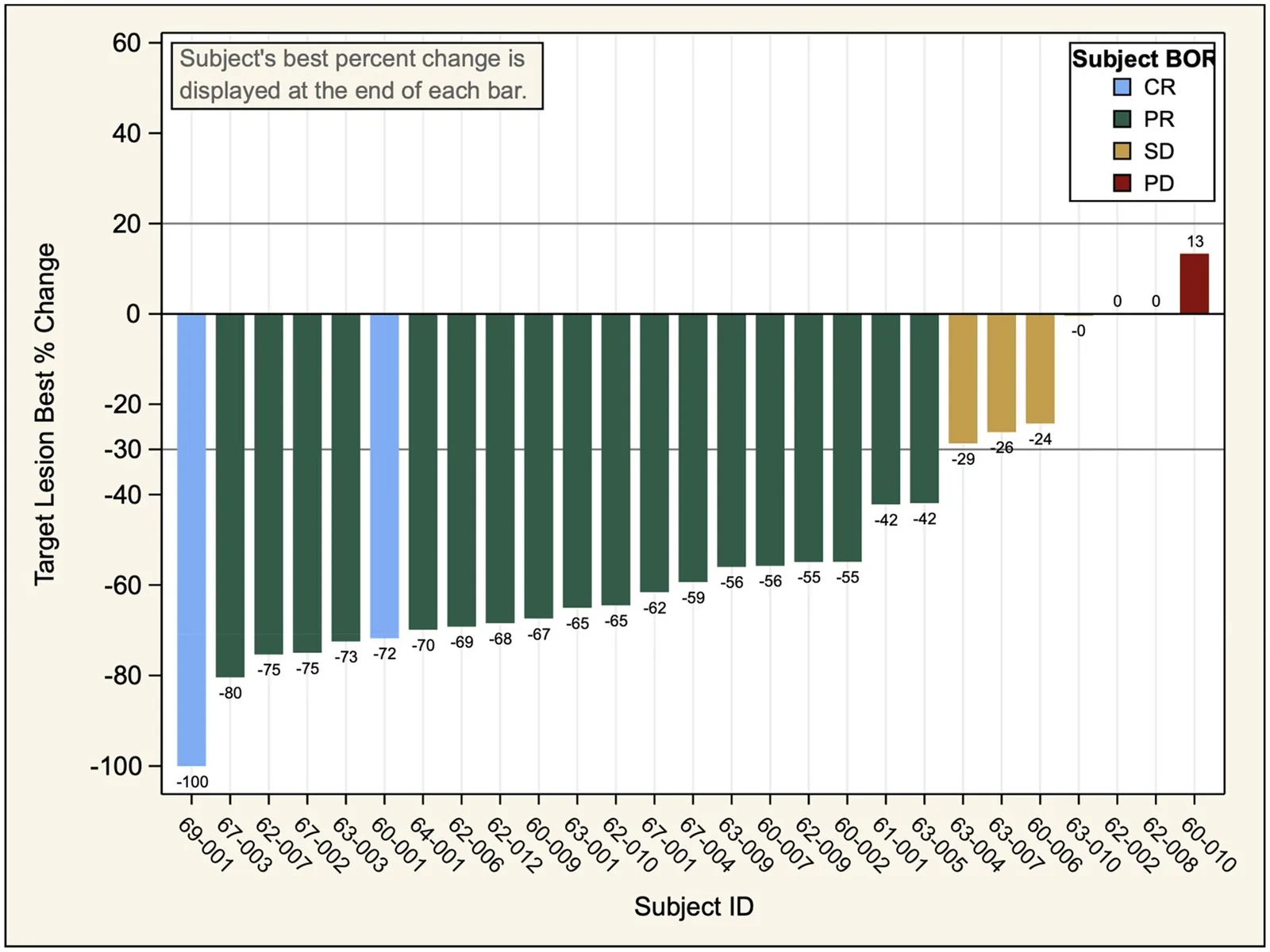
Target lesion best percentage change from baseline by RECiST 1.1.

The primary endpoint was ORR by RECiST 1.1. ORR was 74.1% (20/27) (95% CI, 54-89%, Clopper-Pearson exact binomial), including 2 CRs and 18 PRs. Six patients had stable disease as the best response, including 3 with shrinkage between 20% and 30%. Only one patient had progressed as the best response (with a new lesion on first scan but only growth of +13% from baseline). The disease control rate (DCR) was 96.3% (26/27). The response data are shown above, and the waterfall plot for response is shown in Figure 1.

### PFS, DOR, and OS

**Figure 2.**
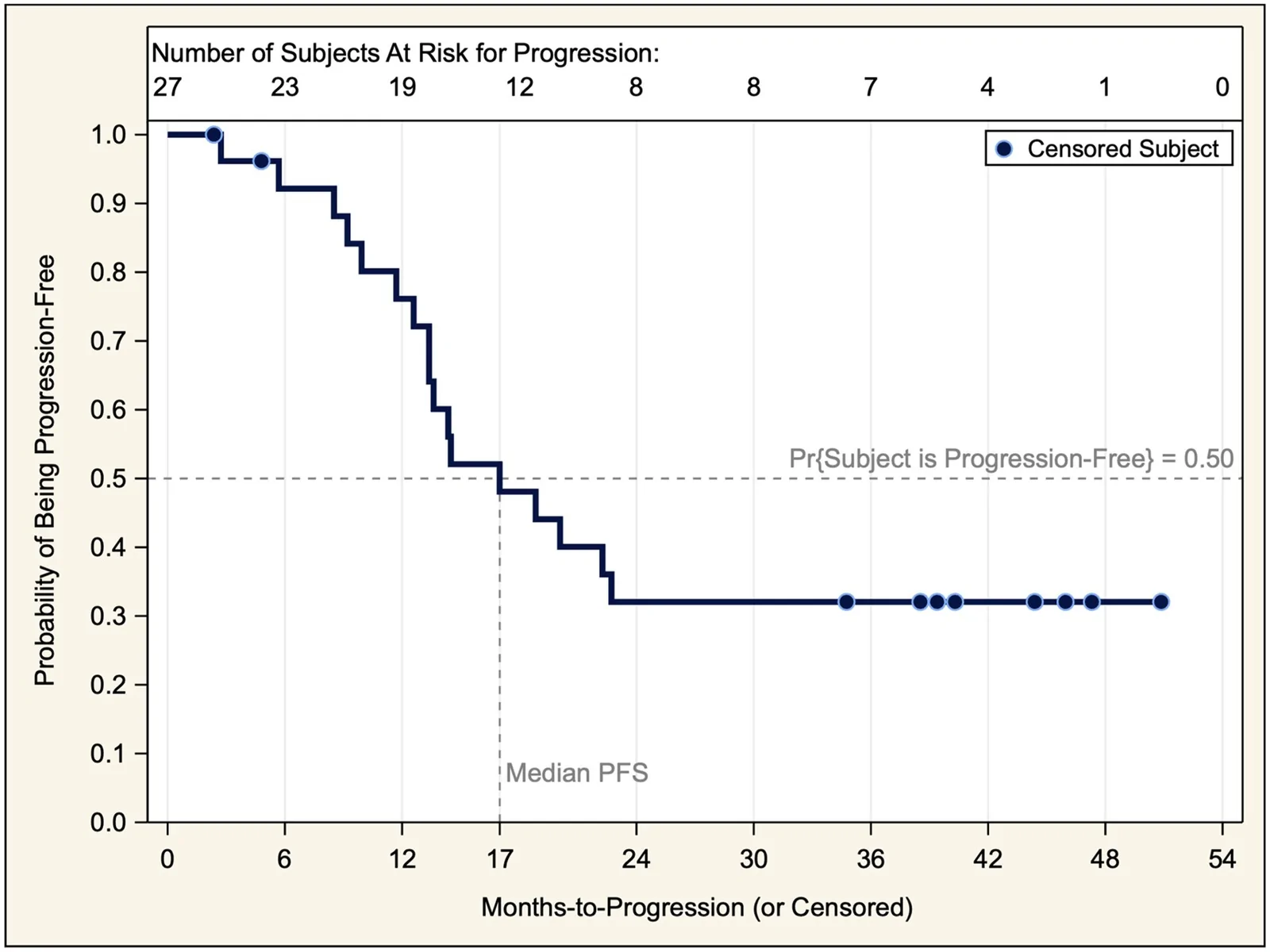
Kaplan-Meier survival curve for progression-free survival (PFS).

**Figure 3.**
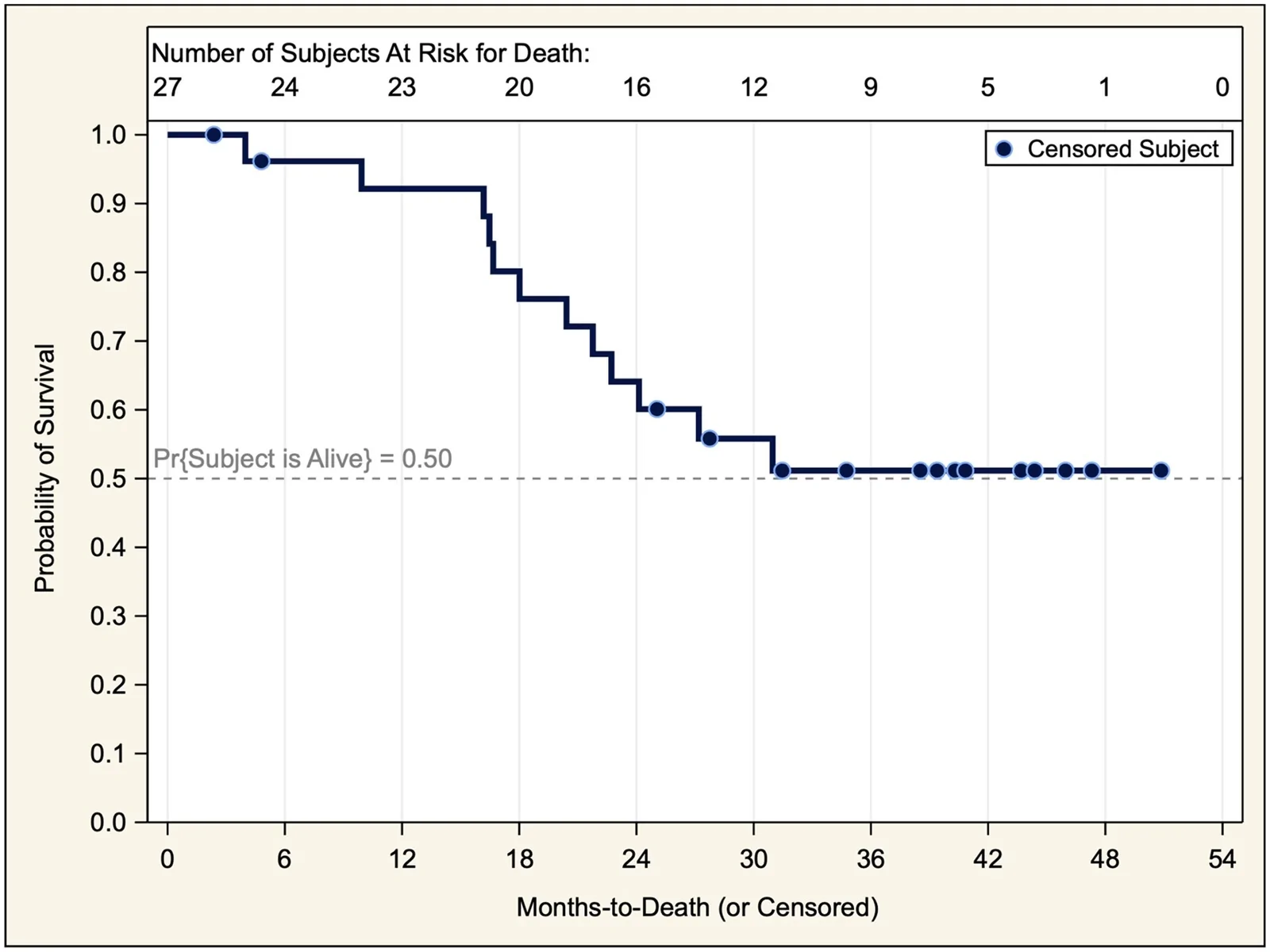
Kaplan-Meier survival curve for overall survival (OS).

The survival function estimates were computed using the Kaplan-Meier method. The median PFS was 17.0 months, with a 95% CI of (13.4, “Infinity”/> 50%) (Figure 2). Duration of response (DOR) for the 20 objective responders was a mean DOR of 20.2 months (range 0.2-48.9). The median OS is not estimable because the survival curve does not cross 0.50 (Figure 3). The Kaplan-Meier survival curve has a final Pr{Survival} value of 51% from the time of the last observed death (31.0 months) until the final censored value of 50.9 months. The restricted mean survival time (mean survival time until the final observed time point) was 35.4 months (SE = 3.3 months). This is an underestimate because it does not account for any potential additional survival beyond 50.9 months for censored patients.

**Table:** 

	SIGNIFICANT ADVERSE EVENTS (Grade ≥3)		
Toxicity	Grade 3 (4)	Total	Percent
**Diarrhea**	10	10	37
**Hypokalemia**	7 (1)	8	30
**Anemia**	6	6	22
**Acneiform rash**	5	5	20
**Hypomagnesemia**	3 (1)	4	15
**Absolute Neutrophil Count (ANC)** **decrease**	3 (1)	4	15
**Nausea**	3	3	11
**Vomiting**	3	3	11
**Abdominal pain**	2	2	7
**Acute kidney injury**	2	2	7
**Dehydration**	2	2	7
**Fatigue**	2	2	7
**Neuropathy**	2	2	7
**Sepsis**	2	2	7
**Thromboembolic event**			

### Toxicity

All subjects experienced a treatment-emergent adverse event (AE) during the trial (see the table above). The highest-grade AE was grade 4 for 4 (15%) subjects, grade 3 for 17 (63%), and grade 2 for 6 (22%). Fifteen (56%) subjects experienced a serious AE (SAE). For grade 3-4 AEs, the most common toxicities were diarrhea, hypokalemia, anemia, and acneiform rash as shown in the accompanying table.

## Discussion

We designed this trial in 2020, as the TRIBE study suggested higher response rates for triplet chemotherapy in first-line therapy of metastatic colon cancer. We wished to combine triplet chemotherapy with optimal antibody therapy in the most appropriate patient population. Randomized phase 3 studies, including Cancer and Leukemia Group B (CALGB)/Southwest Oncology Group (SWOG) 80405 and FIRE-4, as well as a combined analysis, suggested improved OS with the use of anti-EGFR therapy, but the benefits were confined to patients with left-sided, RAS wild-type colon cancers. Furthermore, the PARADIGM trial, conducted in Japan, which compared mFOLFOX plus panitumumab vs bevacizumab in 604 patients with left-sided metastatic colon cancer patients, demonstrated an OS Hazard Ratio (HR) of 0.82, with an 8% increase in response rate with use of panitumumab.^16^

At the time we initiated the study, there were no published US data for FOLFOXIRI with anti-EGFR antibodies. The TRIPLETE randomized trial, from Italian investigators, final report shows an OS benefit for FOLFOXIRI vs FOLFOX with panitumumab (median OS of 41.4 months, HR = 0.79; *P* = 0.049).^17^^,^^18^ In addition, the ORR was 75% for FOLFOXIRI, with a median PFS of 12.7 months. Our multicenter study, though not randomized and considerably smaller, was consistent with an ORR of 74%, a median PFS of 17 months, and a median OS >35 months.

Our positive results may be due to selection bias in choosing patients for this 4-drug regimen at major academic centers, though our investigators attempted to enroll all appropriate patients sequentially. However, we expect similar considerations to enter into patient selection for any randomized trial involving FOLFOXIRI-based therapy in the United States. Given the recent survival data from the larger Italian randomized trial, we believe that the FOLFOXIRI and panitumumab regimen offers survival advantages to patients and should be considered for any patient with left-sided, RAS wild-type CRC undergoing first-line treatment for advanced disease. This survival advantage should be considered in any pharmacoeconomic considerations, as the cost of anti-EGFR therapy is somewhat higher (for a 70 kg individual, about $5880 every 2 weeks compared with $3000 for a bevacizumab biosimilar based on Wholesale Acquisition Costs (WAC)) at the time of publication. With approximately 20% higher median OS, this appears reasonable. These AGICC group data would support further randomized US studies.

## Data Availability

Data regarding this trial are available upon request to the PI, Dr Hochster, or the AGICC Group, Criterium, Inc. (https://www.criteriuminc.com/oncology-consortia/academic-gi-cancer-consortium).
